# Effect of Blood Cadmium Level on Mortality in Patients Undergoing Maintenance Hemodialysis

**DOI:** 10.1097/MD.0000000000001755

**Published:** 2015-10-23

**Authors:** Ching-Wei Hsu, Tzung-Hai Yen, Kuan-Hsing Chen, Dan-Tzu Lin-Tan, Ja-Liang Lin, Cheng-Hao Weng, Wen-Hung Huang

**Affiliations:** From the Department of Nephrology and Division of Clinical Toxicology, Chang Gung Memorial Hospital, Taipei (CWH, THY, KHC, DTLT, JLL, CHW, WHH); Department of Nephrology and Division of Clinical Toxicology, Lin-Kou Medical Center, Taoyuan (CWH, THY, KHC, DTLT, JLL, CHW, WHH); and Chang Gung University and School of Medicine, Taipei, Taiwan, ROC (CWH, THY, KHC, DTLT, JLL, CHW, WHH).

## Abstract

Previous studies of general populations indicated environmental exposure to low-level cadmium increases mortality. However, the effect of cadmium exposure on maintenance hemodialysis (MHD) patients is unclear.

A total of 937 MHD patients from 3 centers in Taiwan were enrolled in this 36-month observational study. Patients were stratified by baseline blood cadmium level (BCL) into 3 groups: high BCL (>0.521 μg/L; n = 312), intermediate BCL (0.286−0.521 μg/L; n = 313), and low BCL (<0.286 μg/L; n = 312). The mortality rates and causes of death were analyzed.

The analytic results demonstrated patients in the high BCL group had a significantly higher prevalence of malnutrition and inflammation than patients in the low and intermediate BCL groups. After 3 years of follow-up, 164 (17.5%) patients died and the major cause of death was cardiovascular disease. A Cox multivariate analysis indicated the high BCL group had increased hazard ratios (HRs) for all-cause mortality (HR = 1.72; 95% confidence interval [CI]: 1.14–2.63; *P* = 0.018), cardiovascular-related mortality (HR = 1.85; 95% CI: 1.09–3.23; *P* = 0.032), and infection-related mortality (HR = 2.27; 95% CI: 1.12–4.55; *P* = 0.035). A Cox multivariate analysis of MHD patients who never smoked (n = 767) indicated the high BCL group had increased HRs for all-cause mortality (HR = 1.67; 95% CI: 1.04–2.63; *P* = 0.048) and cardiovascular-related mortality (HR = 2.08; 95% CI: 1.08–4.00; *P* = 0.044).

In conclusion, BCL is an important determinant of mortality in MHD patients. Therefore, MHD patients should avoid cadmium exposure as much as possible, such as tobacco smoking and eating cadmium-containing foods.

## INTRODUCTION

Cadmium is a well known toxic heavy metal that can cause renal dysfunction, bone disease, cardiovascular disease (CVD), and numerous cancers.^1–3^ In populations exposed to high levels of cadmium, including workers with occupational exposure^4^ and individuals living in heavily polluted areas, there is a link between cadmium exposure and all-cause mortality.^5^ Moreover, 2 recent nationwide follow-up studies^6–7^ of general populations reported environmental exposure to low-level cadmium is associated with increased risk of all-cause and CVD mortality. However, the association between exposure to cadmium and mortality in patients undergoing maintenance hemodialysis (MHD) remains uncertain.

Cadmium can accumulate in patients with end-stage renal disease (ESRD), and MHD patients can have increased blood cadmium levels (BCLs).^8–10^ Moreover, a cross-sectional study showed an elevated BCL was associated with malnutrition and inflammation in MHD patients.^11^ An investigation of a small group of diabetic MHD patients suggested patients with high BCLs had increased risk for all-cause mortality,^12^ but the long-term clinical significance of BCL in MHD patients needs further study. This multicenter 36-month study examined the effect of BCL on mortality in MHD patients.

## METHODS

This clinical study complied with the Declaration of Helsinki and was approved by the Medical Ethics Committee of Chang Gung Memorial Hospital (Taipei, Taiwan). All patients provided informed consent.

## PATIENTS

All patients were recruited from 3 hemodialysis (HD) centers of Chang Gung Memorial Hospital (Taipei, Lin-Kou, and Taoyuan). All enrolled MHD patients were 18 years of age or older and received HD for at least 6 months. Patients with histories of occupational exposure to heavy metals, metal intoxication, or who lived in metal-contaminated areas were excluded. Patients with malignancies, obvious infectious diseases, or who were hospitalized or underwent surgery in the 3 months before enrollment were also excluded.

Most patients were treated with 4-hour HD sessions 3 times per week. HD was conducted using single-use hollow-fiber dialyzers equipped with modified cellulose-based polyamide or polysulfone membranes. In all cases, the dialysate had a standard ion composition in a bicarbonate-based buffer.

CVD was defined as cerebrovascular disease, coronary artery disease, congestive heart failure, or peripheral vascular disease. Hypertension was defined as regular use of an antihypertensive drug to control blood pressure or at least 2 blood pressure measurements above 140/90 mm Hg. Diabetes mellitus was defined by a physician's diagnosis or 2 consecutive tests indicating fasting blood glucose levels >126 mg/dL. Smoking behavior and history of using drugs that could influence the inflammatory state (eg, statins and aspirin) were recorded.

### Blood Levels of Cadmium and Lead

To ensure patients were not exposed to water and dialysate that was contaminated with lead and cadmium during HD, we collected at least 2 samples of water and dialysate from the outlets of the reverse osmosis systems and from the inlets of the dialysate portion of the dialyzers at each HD center using cadmium- and lead-free plastic bottles. Blood cadmium and lead levels were measured as previously described.^13^ Briefly, 900 μL of a modifier solution (NH_4_H_2_PO_4_ + HNO_3_ + Triton X-100) in deionized water and 100 μL of whole blood, or 100 μL of modifier solution and 900 μL of dialysate, were added to a 1.5-mL Eppendorf tube that was immediately shaken. After overnight storage in a refrigerator, the tubes were warmed to room temperature, and then vortexed for 5 to 10 seconds. The diluted sample was transferred to a graphite furnace sampler cup. Cadmium and lead in the acid-digested sample were measured by electro-thermal atomic absorption spectrometry (SpectrAA-200Z; Varian, Palo Alto, CA) with Zeeman's background correction and a L’vov platform. We used internal and external quality control procedures and achieved satisfactory results consistently. A certified commercially prepared product (Seronorm Trace Elements; Sero AS, Billingstads, Norway) was used to determine the intra-batch accuracy and ensure inter-batch standardization. The coefficient of variation of these measurements was 5.0% or less. External quality control was maintained via participation in the National Quality Control Program conducted by the Taiwan government. Patients were divided into 3 equally sized groups for statistical comparisons: a low BCL group (<0.286 μg/L, n = 312), an intermediate BCL group (0.286–0.521 μg/L, n = 313), and a high BCL group (>0.521 μg/L, n = 312).

### Water and Dialysate Cadmium Levels

The cadmium and lead levels of all the water and dialysate samples (n = 12) were less than 0.1 μg/L and less than 2 μg/L, respectively (data not shown). This is far below the American Association for Advancement of Medical Instrumentation (AAMI) standards (cadmium <10 μg/L, lead <50 μg/L).

### Laboratory Parameters

All blood samples were drawn from the arterial end of the vascular access immediately before initiating the midweek HD session, and were then centrifuged and stored at −80°C until analysis. Serum high-sensitivity C-reactive protein (HsCRP) concentrations were measured by immunonephelometry (Nanopia CRP; Daiichi Inc, Tokyo, Japan), with a detection limit of 0.15 mg/L. All other biochemical parameters were measured by standard laboratory procedures with an automatic analyzer. The normalized protein catabolism rate (nPCR) was calculated using validated equations, and was normalized to actual body weight.^14^ The dialysis clearance of urea was measured as described by Daugirdas^15^ and is expressed as Kt/V urea. Serum calcium levels were corrected using the serum albumin levels and the following formula: corrected calcium (mg/dL) = serum calcium (mg/dL) + 0.8 × (4.0 – serum albumin [g/dL]). Cadmium mostly exists in red blood cells, so BCL readings were corrected using hemoglobin levels for men and women, as previously described^16^: Men: corrected BCL (μg/L) = BCL (μg/L) × 14.0/hemoglobin (g/dL); women: corrected BCL (μg/L) = BCL (μg/L) × 12/hemoglobin (g/dL).

### Definition of Malnutrition and Inflammation

We evaluated the effect of BCL on the malnutrition and inflammation status of patients by measurement of serum albumin and HsCRP levels in the 3 BCL subgroups. A serum albumin level less than 3.6 g/dL was defined as malnutrition; this is similar to the lower limit of the normal range in our hospital (3.5 g/dL), and represents the 10th percentile in the Third National Health and Nutrition Examination Survey of Americans.^17,18^ There is no definite HsCRP cutoff level to define the inflammatory state in MHD patients. Thus, we defined the presence of inflammation as an HsCRP level greater than 3 mg/L, a level that correlates with increased cardiovascular risk in the general population.^16,19^

### Follow-Up

Patients were followed for 36 months after the initial assessment. Each death during follow-up was reviewed and assigned an underlying cause by physicians who were unaware of this study. For this analysis, outcomes were categorized as cardiovascular-related deaths, infection-related deaths, or other-cause deaths. Cardiovascular death was defined as an event of arrhythmia, acute or subacute ischemic heart disease, congestive heart failure, intracerebral hemorrhage, occlusion of cerebral arteries, or sudden death. For patients who died in the hospital, information on cardiovascular or infections during follow-up were obtained from discharge diagnosis and death certificates in the charts. For out-of-hospital deaths, family members were interviewed by telephone to fully ascertain the circumstances of the death. The other patients were classified as transferred to other facilities, recipients of renal transplants, transferred to chronic peritoneal dialysis, or remaining on MHD.

### Statistical Analysis

Unless otherwise stated, continuous variables are expressed as means ± standard deviations, and categorical variables as numbers or percentages. Comparisons of the 3 study groups (low, intermediate, and high BCL) were analyzed with trend tests. Log_10_ transformation was used for the following variables that had non-normal distributions: blood lead level, intact parathyroid hormone (iPTH), serum ferritin, BCL, and HsCRP. The Cox proportional hazard model was used to measure all potential variables and determine the significance of variables for prediction of 36-month mortality. The hazard ratio (HR) of death and 95% confidence interval (CI) were obtained by the Cox proportional hazard model. Initially, a univariate Cox model was used to identify the association of all variables with mortality^15,17–19^; then, variables that had *P* values less than 0.05 were entered into the final multivariate Cox model with forward stepwise procedure.

For all statistical tests, a *P* value less than 0.05 was considered significant. The data were analyzed using SPSS software version 12.0 for Windows 95 (SPSS Inc, Chicago, IL).

## RESULTS

### Characteristics of the Study Population

**FIGURE 1:**
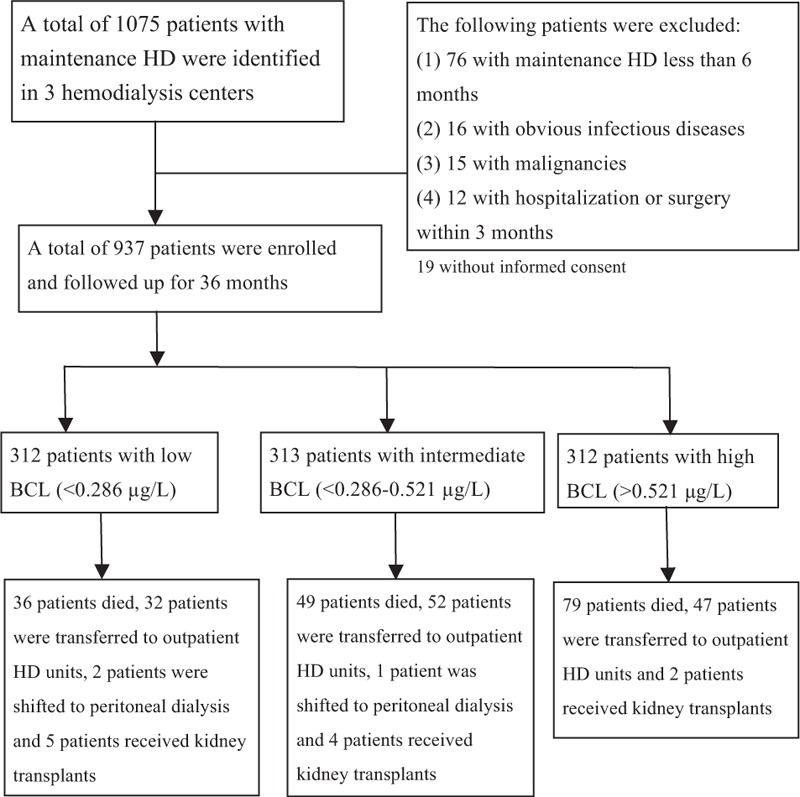
Disposition of maintenance hemodialysis patients during the 36-mo follow-up period.

A total of 937 MHD patients (475 men and 462 women) were enrolled (Fig. 1). The mean patient age was 56.0 ± 13.6 years (range: 8–93 years), median duration of HD was 6.0 years (range: 0.5–26 years), mean BCL was 0.98 ± 1.16 μg/L (range: 0.02–9.53 μg/L), and mean corrected BCL was 1.16 ± 1.45 μg/L (range: 0.02–11.76 μg/L).

Patients were stratified into 3 groups based on BCL: low BCL (<0.286 μg/L, n = 312), intermediate BCL (0.286–0.521 μg/L, n = 313), and high BCL (>0.521 μg/L, n = 312). Table 1 lists the demographic and clinical characteristics of these 3 groups. Patients in the high BCL group were older, had a higher prevalence of diabetes mellitus, and higher levels of body mass index, serum HsCRP, and blood lead, but lower levels of education, serum albumin, creatinine, and iPTH (Table 1). Furthermore, patients in the high BCL group had a significantly higher prevalence of malnutrition (serum albumin <3.6 g/dL) and inflammation (HsCRP >3 mg/L) (Table 1). The groups did not differ significantly in terms of sex, smoking status, history of hypertension and CVD, HD duration, use of a fistula for blood access, use of a biocompatible membrane dialyzer, Kt/V (Daugirdas), nPCR, or residual daily urine less than 100 mL. Moreover, the groups were not statistically different in terms of hemoglobin, transferrin saturation, ferritin, corrected calcium, phosphate, cholesterol, triglyceride, cardiothoracic ratio, presence of the viral hepatitis B antigen, viral hepatitis C antibody, or use of statins and/or aspirin (data not shown).

**TABLE 1:**
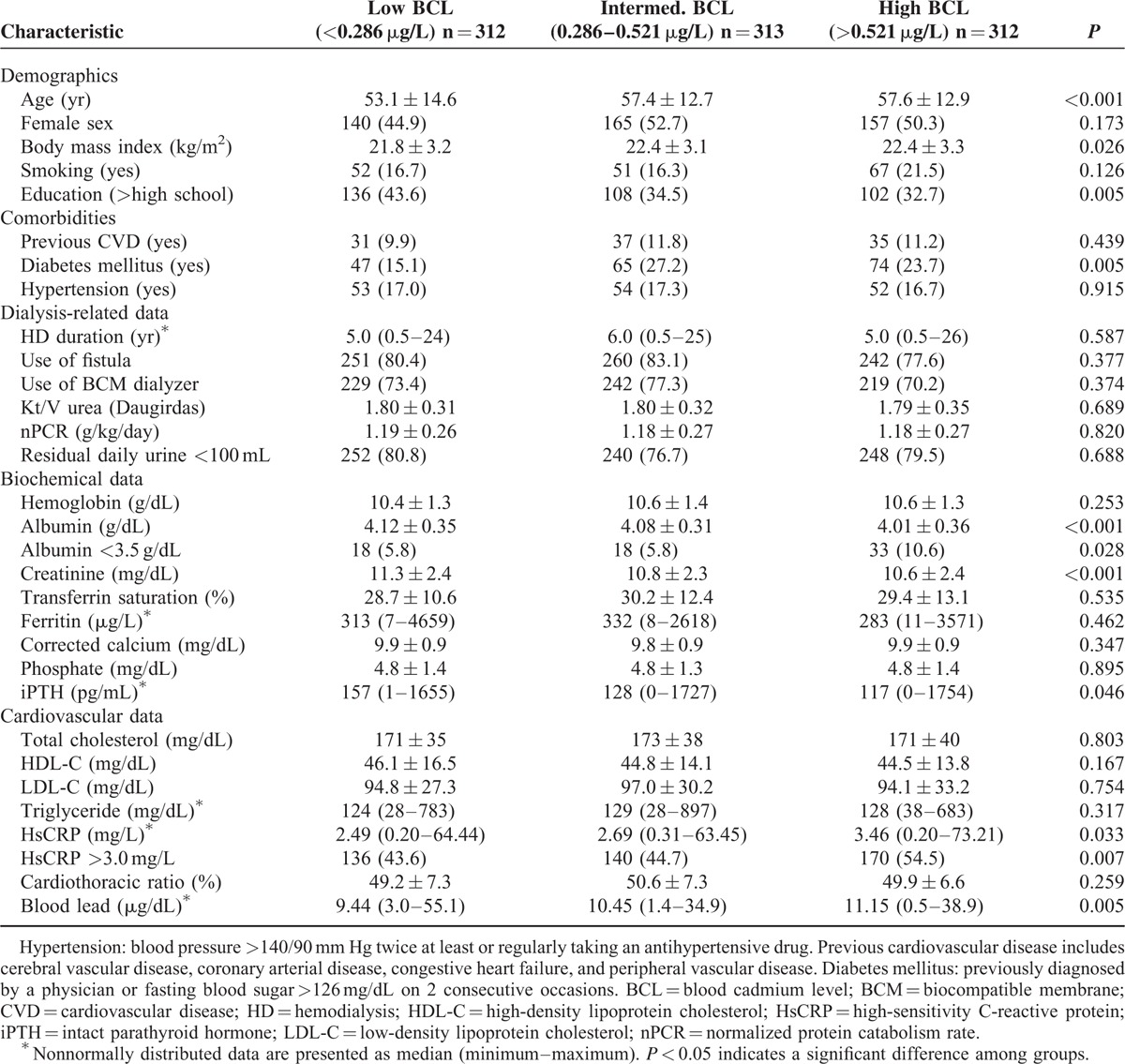
Baseline Characteristics of Patients on Maintenance Hemodialysis, With Stratification by Blood Level of Cadmium (n = 937)

### Analysis of 36-Month Mortality

At the end of the 36-month observation period, 164 of 937 patients (17.5%) died, including 93 from CVD (56.7%), 60 from infection (36.6%), and 11 from unknown causes (6.7%). Among the patients who died from CVD, 50 died from myocardial infarction, 25 from congestive heart failure, 9 from arrhythmia, 7 from stroke, and 2 from other causes. A total of 628 patients completed the 36-month follow-up (Fig. 1).

### Multivariate Cox Proportional Hazards Model for 36-Month Mortality

We used baseline variables that had *P* values less than 0.05 in the univariate analysis for multivariate Cox proportional hazards analysis, with the low BCL group as the reference. The results indicate the high BCL group had increased risk for all-cause mortality (HR: 1.72; 95% CI: 1.14–2.63; *P* = 0.018) (Table 2), cardiovascular-related mortality (HR: 1.85; 95% CI: 1.09–3.23; *P* = 0.032) (Table 3), ischemic heart disease-related mortality (HR: 1.85; 95% CI: 1.01–2.70; *P* = 0.044), and infection-related mortality (HR: 2.27; 95% CI: 1.12–4.55; *P* = 0.035) (Table 4). Analyses using the log_10_ corrected BCL instead of the log_10_ BCL yielded similar results (Tables 2–4).

**TABLE 2:**
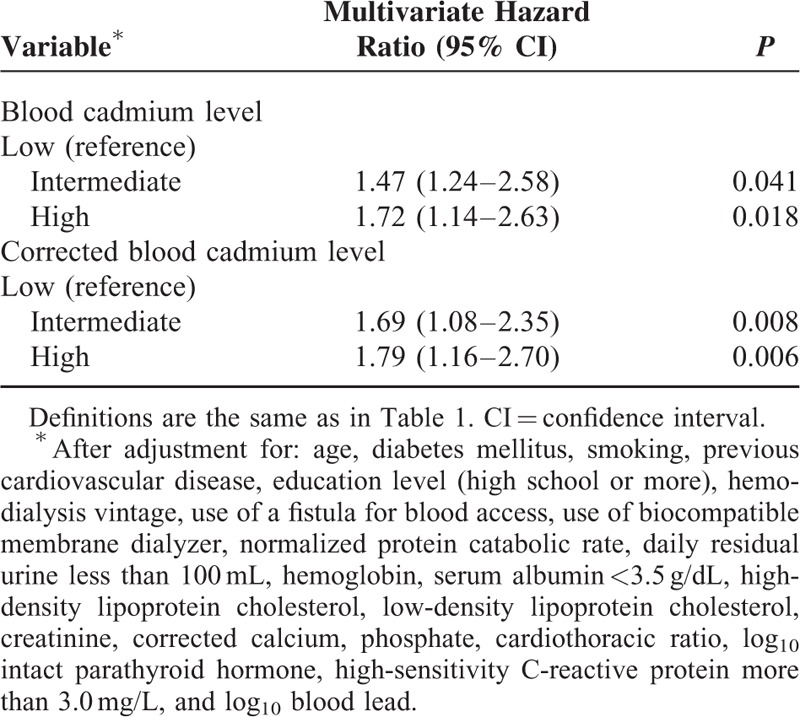
Cox Multivariate Regression Analysis of All-Cause 36-Mo Mortality in Maintenance Hemodialysis Patients According to Baseline Blood Cadmium Level and Corrected Blood Cadmium Level

**TABLE 3:**
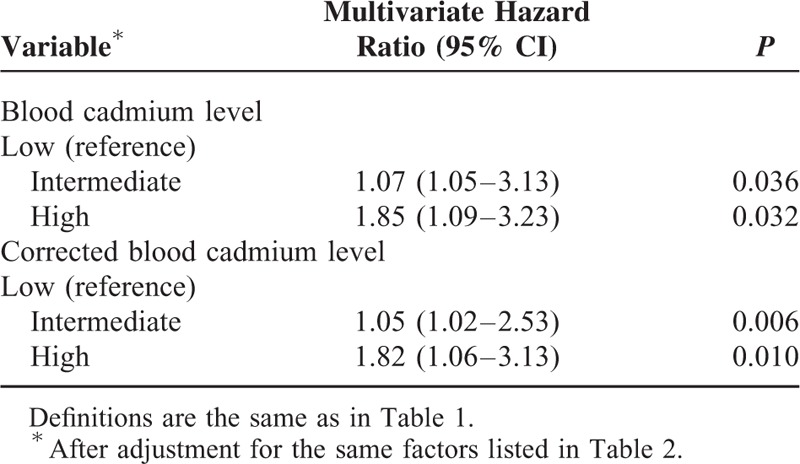
Cox Multivariate Regression Analysis of Cardiovascular-Related 36-Mo Mortality in Maintenance Hemodialysis Patients According to Baseline Blood Cadmium Level and Corrected Blood Cadmium Level

**TABLE 4:**
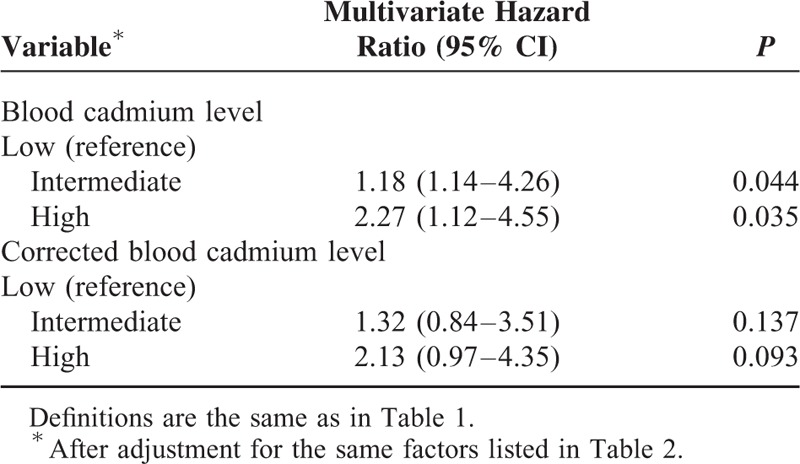
Cox Multivariate Regression Analysis of Infection-Related 36-Mo Mortality in Maintenance Hemodialysis Patients According to Baseline Blood Cadmium Level and Corrected-Blood Cadmium Level

We divided all patients into 2 groups based on smoking status (smoking [n = 170] vs. never smoked [n = 767]), and then performed multivariate Cox proportional hazards analysis. The results indicate nonsmokers in the high BCL group had increased risk for all-cause mortality (HR: 1.67; 95% CI: 1.04–2.63; *P* = 0.048) (Table 5), cardiovascular-related mortality (HR: 2.08; 95% CI: 1.08–4.00; *P* = 0.044) (Table 6) and ischemic heart disease-related mortality (HR: 3.19; 95% CI: 1.23–8.29; *P* = 0.017), using the low BCL group as a reference. However, similar significant risks were not observed in smokers (*P* > 0.05).

**TABLE 5:**
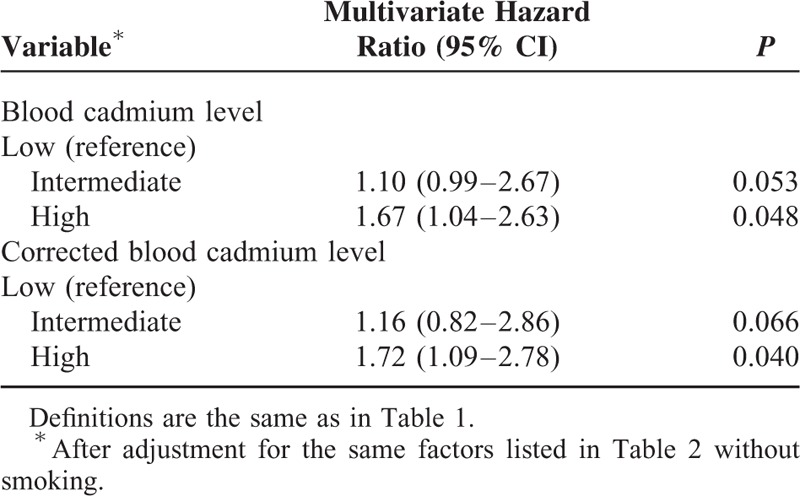
Cox Multivariate Regression Analysis of All-Cause 36-Mo Mortality in Maintenance Hemodialysis Patients Who Never Smoked Tobacco According to Baseline Blood Cadmium Level and Corrected-Blood Cadmium Level

**TABLE 6:**
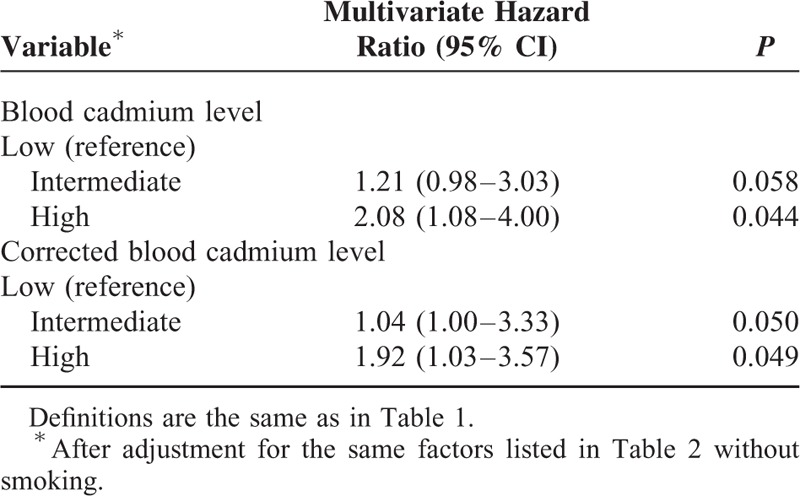
Cox Multivariate Regression Analysis of Cardiovascular-Related 36-Mo Mortality in Maintenance Hemodialysis Patients Who Never Smoked Tobacco According to Baseline Blood Cadmium Level or Corrected-Blood Cadmium Level

We also divided all patients into 2 groups based on the presence of diabetes (diabetes [n = 186] vs. non-diabetes [n = 751]), and then performed a multivariate Cox proportional hazards analysis. These results indicated diabetic patients in the high BCL group had an increased risk for all-cause mortality (HR = 2.83; 95% CI: 1.39 – 5.78; *P* = 0.004), using the low BCL group as the reference. However, a similar significant risk was not observed in non-diabetic patients.

Finally, there were no differences in the baseline data of patients who were lost to follow-up (n = 131) and those not lost to follow-up (n = 806) during the 36-month study period (data not shown).

## DISCUSSION

Our analytic results indicated patients in the high BCL group had lower serum albumin levels (a sign of malnutrition) and higher HsCRP levels (a sign of inflammation) than patients in the intermediate or low BCL groups. These results are similar to those of several previous studies of dialysis patients. For example, a recent study in Japan^20^ demonstrated cadmium accumulation in hair correlated with malnutrition in 60 MHD patients. Our previous study^11^ of 954 MHD patients indicated elevated BCL was associated with poor nutritional status and possible inflammation. Moreover, our study of peritoneal dialysis patients indicated elevated BCL was associated with malnutrition.^21^ Malnutrition and inflammation may predispose ESRD patients to protein-energy wasting and thereby increase the risk of mortality,^18,22^ and this may explain why cadmium exposure increases the risk of mortality in such patients. Therefore, regular BCL measurements may help assess the presence of protein-energy wasting, and may help reduce mortality in MHD patients. However, a further study is required to explore the underlying pathogenesis of cadmium-related mortality.

At the end of our 3-year study, 164 patients had died. After making adjustments for potentially confounding variables, including level of blood lead,^16^ our results indicated the high BCL group had a 1.7-fold increased risk of all-cause mortality, a 1.9-fold increased risk of cardiovascular-related and ischemic heart-related mortality, and a 2.3-fold increased risk of infection-related mortality. In a previous cross-sectional analysis of 954 MHD patients,^11^ we demonstrated an association between increased BCLs with malnutrition and inflammation, but no longitudinal assessment for mortality was performed. In another study of 212 diabetic MHD patients^12^ with 18-month follow-up, we found the association between increased BCLs and all-cause mortality. Compared with the 2 previous studies, although some similar results were obtained, the current study further demonstrated the high BCLs are associated with all-cause, cardiovascular-related, and even infection-related mortality in MHD patients (including diabetic and non-diabetic subjects) with 3-year observational period. This is the first study to demonstrate the high BCL is associated with 36-month mortality in MHD patients. Although these results are similar to those of investigations of general populations,^6–7^ we also found an association of high BCL with infection-related mortality, an association not observed in general populations.^6–7^ This suggests the risk of mortality from cadmium exposure is more severe in MHD patients than in general populations. Since tobacco is the source of cadmium exposure and smoking is a significant cardiovascular risk factor,^1,2^ the confounding effect by smoking must be considered. Remarkably, our multivariate analysis of never smokers indicated elevated BCL was associated with all-cause mortality and CVD and ischemic heart disease-related mortality. This is the first study of MHD patients to report cumulative cadmium exposure is a CVD risk factor independent of tobacco smoking. These results imply environmental cadmium exposure—other than smoking—plays a major role in increasing BCL, and MHD patients should avoid exposures from high-cadmium-containing foods. More studies of other populations are needed to confirm these observations.

The mechanism underlying the association between elevated BCL and mortality in MHD patients remains unknown. However, some previous studies provide several insights into this phenomenon. For example, several animal studies indicated cadmium exposure increases the levels of proinflammatory cytokines,^23–25^ induces lipid peroxidation, and increases oxidative stress in tissues.^26,27^ Additionally, a study in mice further revealed prolonged exposure of low concentration of cadmium triggers proliferation in lung cells and causes severe inflammation.^28^ Similar to animal studies, studies in MHD patients^11,12^ also indicated elevated BCL was associated with inflammation and malnutrition. All of these findings implicate cadmium is correlated with inflammatory status in ESRD patients. Furthermore, inflammation may predispose a dialysis patient to malnutrition.^18,22^ Hence, we may observe there were more malnourished patients in the high BCL group than in the other 2 groups in this study. In addition, inflammation and malnutrition may induce protein-energy wasting, a contributor of infection in ESRD patients.^22^ This may explain why patients in high BCL group had increased risk for infection-related mortality, but not observed in the general population.^6,7^ Moreover, experimental results^29^ indicate cadmium causes endothelial cell dysfunction in vitro and accelerates atherosclerotic plaque formation in vivo. Physiological doses of cadmium increase vascular endothelial permeability by inhibition of endothelial cell proliferation and induction of cell death. Both phenomena are preceded by cadmium-induced DNA strand breaks and a cellular DNA damage response. Another in vitro study indicated Na-K-ATPase is inhibited by cadmium, and that this may play a role in the pathogenesis of renal and cardiovascular damage.^30^ Epidemiological investigations have also shown low-dose cadmium exposure was associated with increased prevalence of peripheral arterial disease^31^ and heart-related diseases,^32^ suggesting cadmium plays a role in the pathogenesis of atherosclerosis. Hence, although elevated BCL increases the risks for cardiovascular-related mortality in MHD patients, further study is needed to clarify the pathogenesis of this effect.

Our results indicate that high BCL is associated with increased mortality in MHD patients, so it is important to identify the sources of cadmium exposure. The level of cadmium in the water and dialysate was less than 0.1 μg/L, far less than the 10 μg/L defined by AAMI standards, so the major source of cadmium exposure is most likely from the environment, including food, drinking water, and air (smoking or passive smoking). Cadmium concentrations are high in certain edible mollusks and crustaceans, such as oysters and other bivalve mollusks, cephalopods, crabs, mushrooms, and in the internal organs of animals, such as the kidney and liver.^1,2^ Therefore, MHD patients should be urged to avoid smoking and cadmium-rich foods to prevent increased BCL because of the nearly complete loss of renal function and the difficulty in removing cadmium from dialysis. Clearly, further studies are needed to determine whether reducing cadmium exposure decreases the mortality of MHD patients.

We also observed diabetic MHD patients had significantly higher BCLs than nondiabetic MHD patients in this study. Moreover, the multivariate Cox analysis of diabetic patients indicated elevated BCL was associated with 36-month all-cause mortality. The result was compatible with our previous report^12^ that BCL is associated with 18-month mortality in 212 diabetic MHD patients. In epidemiological studies, the Third National Health and Nutrition Examination Survey (NHANES III) revealed a significant correlation between elevated urinary cadmium levels and increased fasting blood glucose levels (n = 610), as well as the numbers of individuals diagnosed with type II diabetes (n = 1207) in 8722 examined citizens.^33^ In Belgium, Buchet et al^34^ found a significant association between the urinary biomarkers of renal injury (N-Acetyl-β-d-glucosaminidase and β2-microglobulin), cadmium exposure, and diabetes in a study of 1699 men and women. In animal studies, there was evidence that demonstrated cadmium had diabetogenic effects in both acute and subchronic exposures. For example, Bell et al^35^ found the plasma glucose levels of nonfasted rats became significantly elevated 30 minutes after acute exposure to a single dose of cadmium (0.84 mg/kg, intraperitoneally). In a study of subchronic exposure, Merali et al^36^ revealed rats exhibited significantly elevated fasting blood glucose levels with daily doses of cadmium (1.0 mg/kg) by oral gavage for 45 days. All of these findings suggested cadmium may play a role in the development and progression of diabetes and diabetes-related kidney disease. However, more studies are needed to explore the definite mechanisms of this pathologic effect.

There were some limitations of this study. The enrolled MHD patients were not part of an incident cohort, and this could have caused survival bias. However, the association between elevated BCL and mortality remained after performing an adjustment for HD vintage in the multivariate Cox analysis. Although 131 patients were lost to follow-up during the study period, there were no significant differences of baseline variables between these patients and patients not lost to follow-up. Finally, given the design of this study, with 36 months of follow-up, we could not identify the period over which cadmium exposure occurred. Therefore, additional studies are needed to identify the sources of cadmium exposure in MHD patients who are nonsmokers.

## CONCLUSION

This is the first study to show the high BCL significantly increases the risk of 36-month mortality in MHD patients. The World Health Organization has classified cadmium as a human carcinogen,^1^ and cancer is a common cause of death in ESRD patients.^37^ Our results imply efforts to avoid smoking and foods with high levels of cadmium may reduce mortality in MHD patients. Further studies are required to confirm these observations and to elucidate the pathogenesis of the harmful effects of cadmium.
